# Outcomes of Second-stage Reimplantation After Modular Knee Arthrodesis for Periprosthetic Joint Infection

**DOI:** 10.5435/JAAOSGlobal-D-22-00082

**Published:** 2022-08-11

**Authors:** Alexandra Stavrakis, Erik N. Mayer, Sai Devana, Madhav Chowdhry, Matthew Dipane, Edward McPherson

**Affiliations:** From the Department of Orthopaedic Surgery, University of California Los Angeles, Los Angeles, CA.

## Abstract

Modular knee arthrodesis (MKU) is a salvage treatment for recurrent periprosthetic joint infection (PJI) or PJI associated with notable bone loss. Reimplantation endoprosthetic reconstruction (REI) is an option in patients with MKU who have PJI clearance but are not satisfied with pain or functional outcomes with MKU. The purpose of this study was to evaluate the outcomes of MKU to REI conversion.

**Methods:**

This was a single-center retrospective cohort study of 56 patients who underwent MKU to REI from 2010 to 2019. All patients were staged according to the McPherson staging system. An infecting organism was documented based on pre-MKU aspiration or intraoperative cultures at the time of MKU. Rate ratios were calculated for relevant patient factors. Rate ratios were calculated using Poisson regression with a log link.

**Results:**

The mean REI patient age was 67 years, most of the patients were McPherson B hosts (62.5%) with a type 2 (46.4%) or type 3 (51.8%) limb score, and all PJI were chronic. The most common infecting organisms at the time of MKU were *Staphylococcus epidermidis* (23.2%) and *Staphylococcus aureus* (23.2%, MSSA 14.3%, MRSA 8.9%). The mean time from MKU to REI was 220 days. An 8.9% REI index hospitalization complication rate and a 21.4% overall complication rate (excluding reinfection) were observed. Sixty-seven percent of the patients remained infection-free at an average follow-up of 37 months, among those there was 96.4% implant survivorship. No notable association was observed between index PJI organism or McPherson staging and REI failure secondary to PJI.

**Discussion:**

Approximately two thirds of patients who undergo conversion from MKU to REI have infection-free survival at the midterm follow-up. An index infecting organism and a McPherson host type do not seem to be markedly associated with reinfection risk. These findings help guide expectations of PJI MKU conversion to REI.

Modular knee arthrodesis (MKU), first described in the early 1900s, is an alternative salvage treatment option to above-knee amputation (AKA) for persistent or recurrent knee periprosthetic joint infection (PJI).^1,2,3,4^ Historically, AKA has been associated with lower complication rates and improved outcomes when compared with MKU, although the evidence supporting this is based on small case series or cohorts that also include patients undergoing these treatment options for other indications, in addition to PJI.^5,6,7,8,9^

Although MKU provides knee stability, pain relief, and enhanced mobility, there exists a high rate of patient dissatisfaction due to physical limitations associated with MKU.^10,11,12,13,14^ Consequently, a notable number of patients who show evidence of PJI eradication after MKU seek MKU takedown with conversion to a reimplantation endoprosthetic reconstruction (REI)—a technically challenging procedure with a reported high incidence of complications.^15-17^

Two meta-analyses, both of which comprise several small case series, have shown positive clinical results after conversion of MKU to REI with improved patient satisfaction scores, despite a high risk of postoperative complications including wound dehiscence, extensor mechanism rupture, residual extensor lag, postoperative pain, and recurrent PJI.^18,19^ Currently, there remain a debate among authors and a lack of consensus regarding MKU takedown to REI, with some supporting it given the aforementioned good outcomes, whereas others remain hesitant due to high complication rates.^1,5,20,21,22,23,24,25,26^ The purpose of this study was to evaluate the outcomes of patients who underwent MKU as a salvage treatment for PJI followed by REI at our institution using a standardized technique. Furthermore, we sought to identify any patient or infecting organism characteristics associated with REI failure.

## Methods

**Figure 1:**
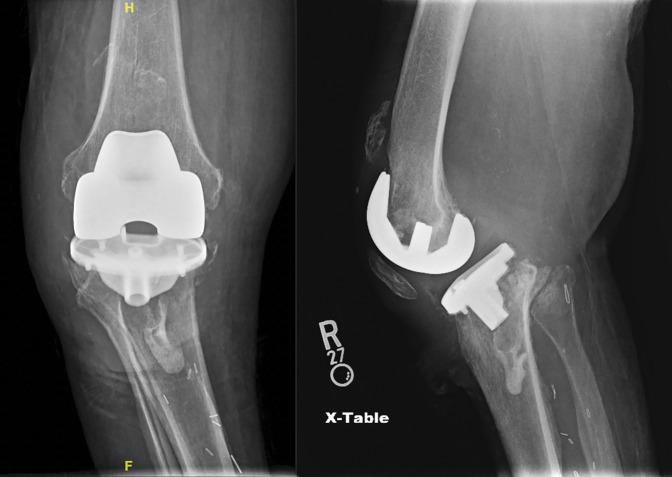
Preoperative AP and lateral views demonstrating the right knee pre-MKU. MKU = modular knee arthrodesis

**Figure 2:**
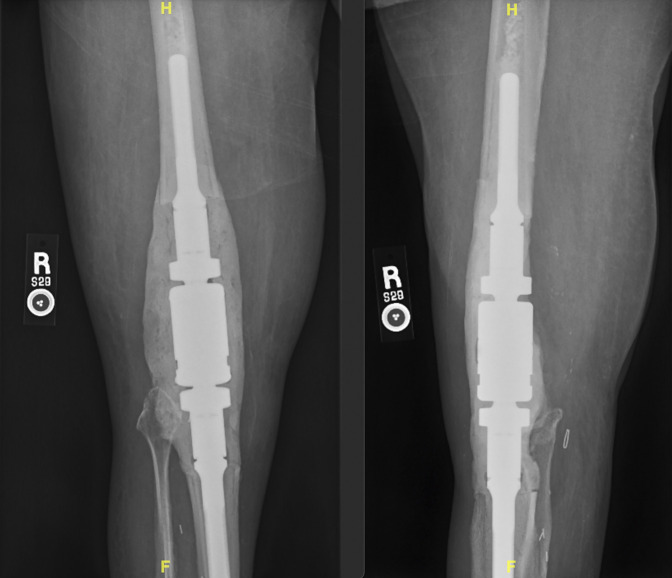
Postoperative AP and lateral views demonstrating the right knee post-MKU. MKU = modular knee arthrodesis

This was a retrospective cohort study that included 56 patients who underwent conversion of MKU to REI at a single institution from January 2010 to December 2019. The mean follow-up was 37 months after REI. All patients had previously undergone MKU specifically for PJI (Figure 1) using a single cemented modular arthrodesis system. The MKU technique involved resection of infected tissue and bone, placement of intramedullary and intra-articular antibiotic eluting beads, and insertion of a cemented endofusion device with a bridging circumferential cement mantle between the femur and the tibia (Figure 2). MKU was done as a salvage alternative to AKA in patients who had failed a two-stage exchange secondary to reinfection or patients with persistent PJI despite an antibiotic cement spacer in place. REI was done in all patients using a single arthroplasty salvage system with distal femoral arthroplasty and/or proximal tibial arthroplasty depending on the degree of bone loss.

Patient characteristics evaluated included patient age, sex, time from MKU to REI, and McPherson staging. An infecting organism was documented based on pre-MKU knee aspiration or intraoperative cultures taken at the time of MKU. REI overall inpatient complications, 90-day medical complications, and surgical complications were recorded. Infection-free survival and overall endoprosthesis survival for other causes were also documented. Medical complications included pneumonia, urinary tract infection, deep vein thrombosis, pulmonary embolism, myocardial infarction, cardiac arrest, cerebrovascular accident, and acute renal failure. Surgical complications were tracked throughout the study period. These included wound dehiscence, superficial surgical site infection, deep surgical site infection, periprosthetic fracture, aseptic loosening, and mechanical failure. Reoperations were also documented and included irrigation and débridement/wound closure, explantation/conversion of REI to MKU, AKA, and open reduction and internal fixation of periprosthetic fracture. We also documented patients who were on chronic oral antibiotic suppression due to failed eradication after an appropriate trial of intravenous antibiotics and were suspected to have persistent infection (continued to have culture-positive knee aspirations).

Extensor mechanism status at the time of REI was documented. Per the senior authors' protocol, for patients with a deficient extensor mechanism, extensor mechanism allograft reconstruction was done at least 1 year after REI given the concern for recurrent infection. No single-stage REI with extensor mechanism reconstructions was done. All were done in a delayed fashion. Subgroup analysis was done for patients who underwent extensor mechanism allograft reconstruction, and extensor mechanism survival was noted at the final follow-up.

Knee Society Scores (KSS) were recorded pre-REI and at most recent follow-up post-REI. Knee range of motion at the final follow-up was documented. SAS statistical analysis software (SAS Institute) was used to calculate rate ratios for relevant patient factors. Rate ratios were calculated using Poisson regression with a log link. Statistical significance was defined as *P* < 0.05. This study was approved by the Institutional Review Board.

## Results

The mean patient age was 67 years (range: 45 to 90 years), and 50% of the patients were female. The mean time from MKU to REI was 220 days (range: 44 to 1582 days). Most of the patients were McPherson B hosts (A: 17.9%; B: 62.5%; and C: 19.6%) and had type 2 or 3 extremity (1: 1.8%; 2: 46.4%; and 3: 51.8%). All patients had a chronic PJI at the time of MKU (>4 weeks) (Table 1). The most common PJI organisms at the time of MKU were *S epidermidis* (23.2%), methicillin-sensitive *S aureus* (14.3%), and methicillin-resistant *S aureus* (8.9%) (Table 2).

**Table 1:** Patient Characteristics

Age (yr)	67 ± 7.8 (45-90)
Sex (%)	
Female	50
Male	50
Time MKU to REI (mean days)	220 ± 285 (94-1582)
McPherson host staging system (%)	
Host	
A	17.9
B	62.5
C	19.6
Extremity	
1	1.8
2	46.4
3	51.8
Infection type	
I	0
II	0
III	100

**Table 2:** Organism Characteristics

Organism	%
*Staphylococcus epidermidis*	23.2
MSSA	14.3
MRSA	8.9
Multiorganism	7.1
*Escherichia coli*	5.4
*Enterococcus faecalis*	3.6
*Candida*	3.6
*Pseudomonas aeruginosa*	1.8
Other	19.6
Culture-negative	12.5

After REI, there was an 8.9% inpatient complication rate. Twenty-one percent of the patients sustained at least one complication throughout the study period, excluding reinfection; 33.3% of complications were medical; and 66.7% were surgical (Table 3).

**Table 3:** Patient Outcomes

Complications/Outcomes	(%)
In-house complications (%)	8.9
Overall complications (%, excluding reinfection)	21.4
Medical	33.3
Surgical	66.7
Revision surgery (%)	41.1
Irrigation and débridement, wound closure	8.9
Explant/conversion to fusion	16.1
Amputation	12.5
Periprosthetic fracture	3.6
Chronic antibiotic suppression (%)	5.4
Infection-free survival (%)	66.1
Survivorship of endoprosthesis (excluding reinfection) (%)	96.4
Extensor mechanism status	
Intact (%)	46.4
Deficient (%)	53.6
Delayed extensor mechanism allograft	16.7
Extensor mechanism allograft survival	60

By the final follow-up, there was a 41.1% revision surgery rate, which was subdivided by the following: irrigation and débridement with wound closure for superficial wound dehiscence (8.9%), explantation and reconversion to MKU for recurrent PJI (16.1%), AKA for recurrent PJI (12.5%), and open reduction and internal fixation or revision arthroplasty for periprosthetic fracture (3.6%). Chronic antibiotic suppression therapy was used in 5.4% of the patients. Overall, 66.1% of the patients had infection-free survival at the final follow-up. This did not include those who remained on chronic antibiotic suppression therapy. When excluding reinfections, overall endoprosthesis survivorship was 96.4%. Both cases of noninfectious failure were due to fracture. The mean knee arc of motion at the final follow-up was 95° ± 25°.

At the time of REI, the extensor mechanism was intact in 46.4% of the patients. In the group of patients with an insufficient extensor mechanism, 16.7% underwent delayed extensor mechanism allograft reconstruction and 60% had survival of the extensor mechanism allograft at the final follow-up (one patient failed secondary to infection and the other secondary to rupture of the allograft) (Table 3).

Both clinical and functional mean KSS significantly improved after REI (clinical KSS: 28-52 ± 21, *P* < 0.05; functional KSS: 19-41, *P* < 0.05) (Table 4). No statistically significant association was observed between either the McPherson host staging system or the PJI infecting organism type and REI failure secondary to PJI recurrence (Tables 5 and 6).

**Table 4:** Knee Society Scores

Mean	Preconversion	Postconversion	*P* Value
Clinical	28 ± 15 (10-65)	52 ± 21 (20-92)	<0.05
Functional	19 ± 8 (0-50)	41 ± 16 (0-90)	<0.05

**Table 5:** Reinfection Risk by Organism

Organism	Failure % (95% CI)	RR (95% CI)	*P* Value
Other	18.2 (0.0, 43.4)	1	
Staph epi	30.8 (0.6, 60.9)	1.41 (0.34, 5.90)	0.6378
Staph aureus	50.0 (1.0, 99.0)	2.75 (0.69, 11.00)	0.1525
Culture-neg	14.3 (0.0, 42.3)	1.05 (0.18, 6.27)	0.9594
MRSA	60.0 (0.0, 100.0)	2.93 (0.66, 13.11)	0.1588
Multiorganism	50.0 (0.0, 100.0)	1.83 (0.31, 10.97)	0.5067
*E coli*	33.3 (0.0, 98.7)	1.22 (0.13, 11.75)	0.8620
*Candida albicans*	0.0	—	—
*Enterococcus*	50.0 (0.0, 100.0)	1.83 (0.19, 17.62)	0.5996
*Pseudomonas*	100.0	3.67 (0.38, 35.25)	0.2605

**Table 6:** Reinfection Risk by McPherson Host Type

Grade	Failure % (95% CI)	RR (95% CI)	*P* Value
A	36.4 (0.7, 72.0)	1.33 (0.30, 5.96)	0.7064
B	35.3 (15.3, 55.3)	1.29 (0.37, 4.59)	0.6896
C	27.3 (0.0, 58.1)	1	—
Extremity			
1	100.0	4.29 (0.53, 34.83)	0.1734
2	23.3 (6.0, 40.6)	1	
3	44.0 (18.0, 70.0)	1.89 (0.73, 4.86)	0.1895

## Discussion

Given that REI after MKU initially performed for persistent or recurrent PJI is a relatively rare procedure, the perioperative and long-term outcomes are not well known. To our knowledge, this represents the largest single-center study evaluating the outcomes of patients who undergo conversion of MKU to REI. It is also the largest study to evaluate these outcomes specifically in patients who initially underwent MKU as a salvage treatment alternative to AKA for PJI, excluding those undergoing MKU for other indications such as oncology-related or trauma-related salvage procedures.

Jauregui et al, who performed a meta-analysis that included 10 studies with a total of 98 patients, reported slightly higher complications than those observed in this study. There were an overall 47% complication rate, 25% arthroplasty revision rate, and overall failure rate of 11%. Notably, patients included in the meta-analysis by Jauregui et al^18^ underwent MKU for various indications, not exclusively for PJI; therefore, the patient population is slightly different from ours. A meta-analysis by Kernkamp et al, which included six studies with 123 patients, showed similar knee range-of-motion improvements to those seen in our study (mean 80° vs mean 95° in our study). They also reported a notable improvement in patient clinical and functional outcome scores after REI. Although there was a lower infection rate noted compared with that in our study (11% in the work of Kernkamp et al), the meta-analyses of Kernkamp et al^19^ and Jauregui et al included patients who underwent index MKU nonexclusively for PJI, which may explain the higher rate of infection in our results, given that all patients had a history of infection. It is unclear if this higher infection rate seen in our study is related to an indolent infection undetected during the pre-REI infection workup or to specific host factors such as comorbidities or McPherson host type or extremity grade that may predispose patients to infection.

There are several limitations to this study. It is retrospective in nature, and there is no comparison group. All patients underwent index MKU and REI using the same implant system; therefore, these outcomes may not be directly comparable with those who undergo either MKU or REI using other implant systems or surgical techniques. For example, the technique for index MKU may play a role in patient outcomes after REI. One advantage of the MKU technique used in this study is that it allows for radical débridement of bone and the surrounding soft tissues that may otherwise be a nidus for infection because fusion is dependent on initial implant cementation and does not rely on bony compression such is required with fusion obtained with the use of other techniques such as plating or external fixation/Ilizarov/Taylor spatial frame methods. The cementing technique used with this MKU technique also allows for direct high-dose antibiotic elution by the use of antibiotic cement for fixation. Similarly, given the large amount of bone resection inherent to the technique of index MKU used in this study, REI requires endoprosthetic reconstruction with a distal femoral arthroplasty, and in some cases proximal tibial arthroplasty. It is not clear how this may affect long-term aseptic loosening rates.

Despite these limitations, this represents the largest single-center cohort of patients undergoing conversion of PJI-indicated MKU to REI. The findings in this study serve us to guide arthroplasty surgeons and their MKU patients with the expected overall complication rate, and specifically the risk of PJI recurrence when considering REI conversion. Additional studies are needed to determine the long-term outcomes of this patient population, in particular both the long-term infection-free and aseptic loosening survival of REI.
